# Supplementary material for: Physical interpretation of non-normalizable harmonic oscillator states and relaxation to pilot-wave equilibrium
Source: Sci Rep. 2024 Jan 13;14:669. doi: 10.1038/s41598-023-50814-w (PMC10787783; doi:10.1038/s41598-023-50814-w)
Supplement: Supplementary file 1 — Supplementary Information 1. [file 41598_2023_50814_MOESM1_ESM.pdf]

Physical interpretation of non-normalizable harmonic oscillator states and relaxation to pilot-wave equilibrium

Indrajit Sen*
Institute for Quantum Studies, Chapman University
One University Drive, Orange, CA, 92866, USA

Action of ladder operators on non-normalizable eigenstates

In orthodox quantum mechanics, lowering and raising operators provide an alternative method to obtain the quantized energy levels and eigenstates of the harmonic oscillator. It is useful to discuss the action of these operators on the non-normalizable harmonic oscillator eigenstates. We recall that the operators are defined as

$$\hat{a} = \frac{1}{\sqrt{2}}(\frac{d}{dy} + y) \tag{1}$$

$$\hat{a}^\dagger = \frac{1}{\sqrt{2}}(-\frac{d}{dy} + y) \tag{2}$$

where $\hat{a}$ ($\hat{a}^\dagger$) is the lowering (raising) operator. It is convenient here to work with the closed form expressions of $\varphi_0^K(y)$ and $\varphi_0^K(y)$ in terms of confluent hypergeometric functions of the first kind.

Action of lowering operator

Let us first consider the action of $\hat{a}$ on $\varphi_0^K(y)$:

$$\begin{aligned}
\hat{a}\varphi_0^K(y) &= e^{-y^2/2}\frac{dh_0^K(y)}{dy} &(3)\\
&= e^{-y^2/2}\frac{dM(\frac{1-K}{4},\frac{1}{2},y^2)}{dy} &(4)\\
&= e^{-y^2/2}(1-K)yM(\frac{3-(K-2)}{4},\frac{3}{2},y^2) &(5)\\
&= (1-K)\varphi_1^{K-2}(y) &(6)
\end{aligned}$$

where, in equation (5), we have used the identity

$$\frac{d}{dx}M(a,b,x) = \frac{a}{b}M(a+1,b+1,x) \tag{7}$$

with $x = y^2$, $a = (1-K)/4$ and $b = 1/2$.

Similarly, we can evaluate the action of $\hat{a}$ on $\varphi_1^K(y)$ as follows:

$$\begin{aligned}
\hat{a}\varphi_1^K(y) &= e^{-y^2/2}\frac{dh_1^K(y)}{dy} &(8)\\
&= e^{-y^2/2}\frac{d\big(yM(\frac{3-K}{4},\frac{3}{2},y^2)\big)}{dy} &(9)\\
&= e^{-y^2/2}M(\frac{1-(K-2)}{4},\frac{1}{2},y^2) &(10)\\
&= \varphi_0^{K-2}(y) &(11)
\end{aligned}$$

*Electronic address: isen@chapman.edu

where, in equation (10), we have used the identity

$$\frac{d}{dx}\big(x^{b-1}M(a,b,x)\big) = (b-1)x^{b-2}M(a,b-1,x) \tag{12}$$

with $x = y^2$, $a = (3-K)/4$ and $b = 3/2$.

Equation (6) confirms that the ground state in orthodox quantum mechanics $\varphi_0^{K=1}(y)$ is annihilated by $\hat{a}$. However, it is interesting to note that $\varphi_0^{K=-1}(y)$ and $\varphi_1^{K=-1}(y)$ exist nevertheless – they are just non normalizable. In fact, equation (11) implies that $\varphi_1^{K=1}(y)$ is transformed to $\varphi_0^{K=-1}(y)$ as a result of the action of $\hat{a}$.

Using (6) and (11), we have

$$\hat{a}\psi_{\theta,\phi}^K(y) = \cos\theta\hat{a}\varphi_0^K(y) + \sin\theta e^{i\phi}\hat{a}\varphi_1^K(y) \tag{13}$$

$$= \cos\theta(1-K)\varphi_1^{K-2}(y) + \sin\theta e^{i\phi}\varphi_0^{K-2}(y) \tag{14}$$

$$= \cos\theta_{K-}\varphi_0^{K-2}(y) + \sin\theta_{K-}e^{i(2\pi-\phi)}\varphi_1^{K-2}(y) = \psi_{\theta_{K-},2\pi-\phi}^{K-2}(y) \tag{15}$$

where $\cos\theta_{K-} = \sin\theta/\sqrt{\cos^2\theta(1-K)^2+\sin^2\theta}$ and $\sin\theta_{K-} = \cos\theta(1-K)/\sqrt{\cos^2\theta(1-K)^2+\sin^2\theta}$. Equation (15) implies that the action of $\hat{a}$ on $\psi_{\theta,\phi}^K(y)$ is to generate $\psi_{\theta_{K-},2\pi-\phi}^{K-2}(y)$.

Action of raising operator

Let us consider the action of $\hat{a}^\dagger$ on φ_0^K.

$$\hat{a}^\dagger\varphi_0^K(y) = e^{-y^2/2}(2yh_0^K(y) - \frac{dh_0^K(y)}{dy}) \tag{16}$$

$$= e^{-y^2/2}(2yM(\frac{1-K}{4},\frac{1}{2},y^2) - \frac{dM(\frac{1-K}{4},\frac{1}{2},y^2)}{dy}) \tag{17}$$

$$= e^{-y^2/2}(1+K)yM(\frac{3-(K+2)}{4},\frac{3}{2},y^2) \tag{18}$$

$$= (1+K)\varphi_1^{K+2}(y) \tag{19}$$

where, in equation (18), we have used the identity

$$\frac{d}{dx}\big(e^{-x}M(a,b,x)\big) = \frac{a-b}{b}e^{-x}M(a,b+1,x) \tag{20}$$

with $x = y^2$, $a = (1-K)/4$ and $b = 1/2$.

Similarly, we can evaluate the action of $\hat{a}^\dagger$ on φ_1^K as follows:

$$\hat{a}^\dagger\varphi_1^K(y) = e^{-y^2/2}(2yh_1^K(y) - \frac{dh_1^K(y)}{dy}) \tag{21}$$

$$= e^{-y^2/2}(2y^2M(\frac{3-K}{4},\frac{3}{2},y^2) - \frac{d\big(yM(\frac{3-K}{4},\frac{3}{2},y^2)\big)}{dy}) \tag{22}$$

$$= -e^{-y^2/2}M(\frac{1-(K+2)}{4},\frac{1}{2},y^2) \tag{23}$$

$$= -\varphi_0^{K+2}(y) \tag{24}$$

where, in equation (23), we have used the identity

$$\frac{d}{dx}\big(e^{-x}x^{b-1}M(a,b,x)\big) = (b-1)x^{b-2}M(a-1,b-1,x) \tag{25}$$

with $x = y^2$, $a = (3-K)/4$ and $b = 3/2$.

Note that equation (19) implies that $\varphi_0^{K=-1}$ is annihilated upon being acted by $\hat{a}^\dagger$. This is similar to the annihilation of the ground state $\varphi_0^{K=+1}$ upon being acted by $\hat{a}$.

Using (19) and (24), we have

$$\hat{a}^\dagger\psi^K_{\theta,\phi}(y) = \cos\theta\hat{a}^\dagger\varphi_0^K(y) + \sin\theta e^{i\phi}\hat{a}^\dagger\varphi_1^K(y) \tag{26}$$

$$= \cos\theta(1+K)\varphi_1^{K+2}(y) + \sin\theta e^{i(\phi+\pi)}\varphi_0^{K+2}(y) \tag{27}$$

$$= \cos\theta_{K+}\varphi_0^{K+2}(y) + \sin\theta_{K+}e^{i(\pi-\phi)}\varphi_1^{K+2}(y) = \psi^{K+2}_{\theta_{K+},\pi-\phi}(y) \tag{28}$$

where $\cos\theta_{K+} = \sin\theta/\sqrt{\cos^2\theta(1+K)^2+\sin^2\theta}$, $\sin\theta_{K+} = \cos\theta(1+K)/\sqrt{\cos^2\theta(1+K)^2+\sin^2\theta}$. Similar to equation (15), we see from (28) that the action of $\hat{a}^\dagger$ on $\psi^K_{\theta,\phi}(y)$ is to generate $\psi^{K+2}_{\theta_{K+},\pi-\phi}(y)$.

From equations (6), (11), (19) and (24), we see that $\hat{a}$ and $\hat{a}^\dagger$ continue to act as lowering and raising operators respectively in the general scenario where normalizability is violated. However, unlike in the normalizable scenario, it is not possible to obtain all the quantum states by successively using $\hat{a}$ and $\hat{a}^\dagger$ on a single eigenstate corresponding to a single K. This is because, firstly, K can take values from $(-\infty,+\infty)$ once normalizability is dropped. Secondly, equations (6) and (19) imply that $\hat{a}\varphi_0^{K=1}$ and $\hat{a}^\dagger\varphi_0^{K=-1}$ are zero. Thirdly, the eigenstates are doubly degenerate at any value of K. Therefore, although both the analytic method and ladder operator approach are equivalent in orthodox quantum mechanics, the analytic method turns out to be more general when the normalizability assumption is dropped.

Approximate form of the eigenstate at large $\pm y$

Consider a harmonic-oscillator eigenstate $\psi^K(y)$. We know from the main text that

$$\psi^K(y) = e^{-y^2/2}\left[a_0 M(\frac{1}{4}(1-K),\frac{1}{2},y^2) + a_1 y M(\frac{1}{4}(3-K),\frac{3}{2},y^2)\right] \tag{29}$$

Using the asymptotic form [1] of $M(c,d,y)$ as $y\to\infty$

$$M(c,d,y) \sim \frac{e^y y^{c-d}}{\Gamma(c)}\sum_{s=0}^{\infty}\frac{(1-c)_s(d-c)_s}{s!}y^{-s} \tag{30}$$

valid for $c \neq 0,-1,-2...$, we find that

$$M(\frac{1}{4}(1-K),\frac{1}{2},y^2) = \frac{e^{y^2}y^{-\frac{1+K}{2}}}{\Gamma(\frac{1-K}{4})}\{1+\frac{(3+K)(1+K)}{16y^2}\} \tag{31}$$

$$yM(\frac{1}{4}(3-K),\frac{3}{2},y^2) = \frac{e^{y^2}y^{-\frac{1+K}{2}}}{\Gamma(\frac{3-K}{4})}\{1+\frac{(3+K)(1+K)}{16y^2}\} \tag{32}$$

where we have retained terms only up to $1/y^2$. Note that $K = 1+4n$ ($K = 3+4n$) for $M(\frac{1}{4}(1-K),\frac{1}{2},y^2)$ $\big(M(\frac{1}{4}(3-K),\frac{3}{2},y^2)\big)$ if $c = 0,-1,-2...$ in $M(c,d,y)$. For these values of K, $M(c(K),d,y)$ is a polynomial of finite order as the power series terminates, and $e^{-y^2/2}M(c,d,y)$ vanishes at $|y|\to\infty$. Therefore, we need only concern ourselves with the non-normalizable part of $\psi^K(y)$ to determine its asymptotic behaviour. Using (31), (32), the asymptotic form of the quantum state can be written as

$$\lim_{|y|\to\infty}\psi^K(y) = e^{\frac{y^2}{2}}y^{-\frac{1+K}{2}}\{1+\frac{(3+K)(1+K)}{16y^2}\} \tag{33}$$

where have eliminated the global phase and magnitude.

[1] *NIST Digital Library of Mathematical Functions.* `https://dlmf.nist.gov/`, Release 1.1.10 of 2023-06-15. F. W. J. Olver, A. B. Olde Daalhuis, D. W. Lozier, B. I. Schneider, R. F. Boisvert, C. W. Clark, B. R. Miller, B. V. Saunders, H. S. Cohl, and M. A. McClain, eds.
